# Supplementary figures and images for: Seroprevalence of dengue virus antibodies among multiple species of non-human primates in Senegal suggests that sylvatic dengue virus is maintained in non-primate reservoirs in this region
Source: PLoS Negl Trop Dis. 2026 Jan 27;20(1):e0013946. doi: 10.1371/journal.pntd.0013946 (PMC12863672; doi:10.1371/journal.pntd.0013946)

Chlorocebus sabaues

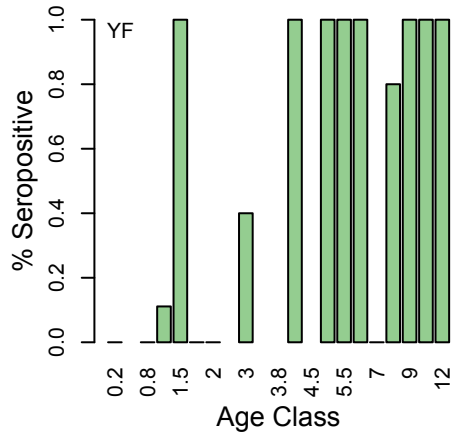

Papio papio

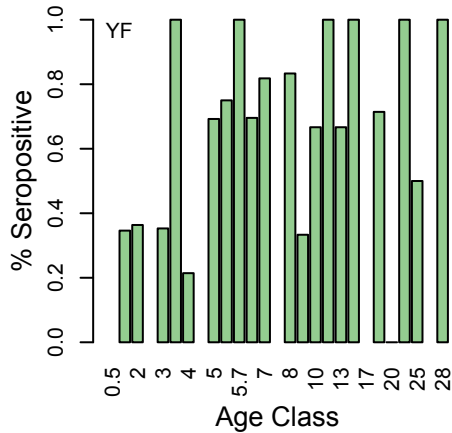

Erythrocebus patas

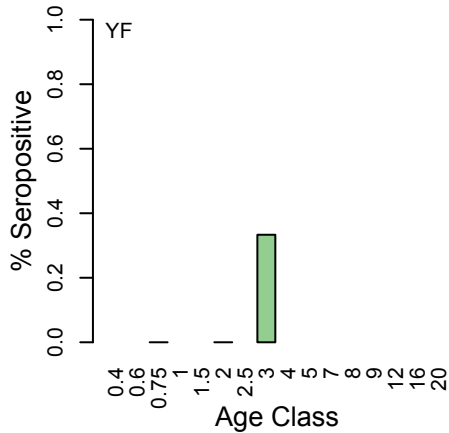

Supplement: S1 Fig — (PDF) [file pntd.0013946.s001.pdf]

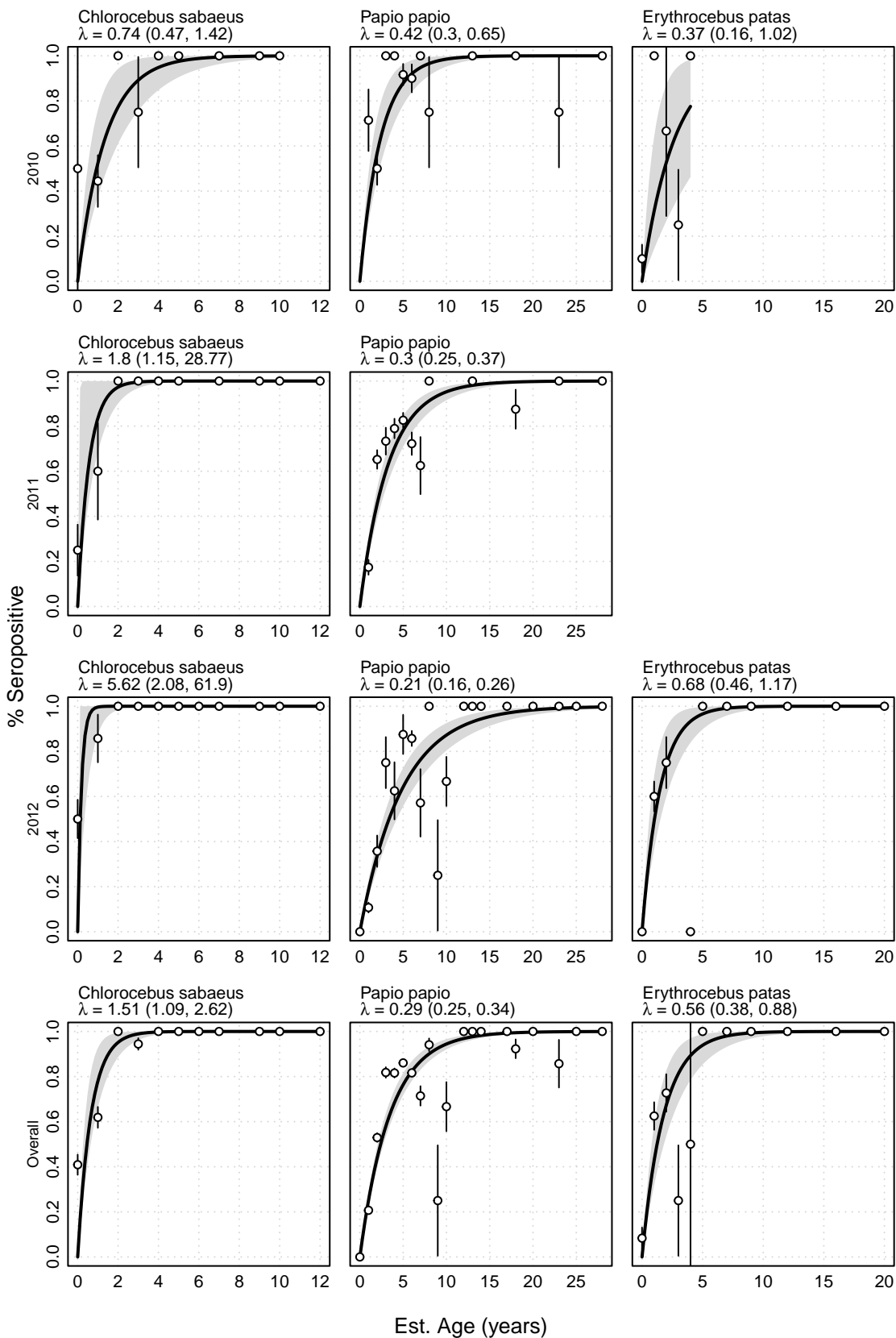

Supplement: S2 Fig — Points represent the proportion of seropositive animals per age and year, with associated confidence interval as vertical lines. Seroprevalence estimated from model using best fitting forces of infection (black line), with grey shaded bands representing bootstrap 95% confidence intervals. DENV PRNT50 seropositivity was used to define prior infection. (PDF) [file pntd.0013946.s002.pdf]
