# Supplementary material for: Seroprevalence of dengue virus antibodies among multiple species of non-human primates in Senegal suggests that sylvatic dengue virus is maintained in non-primate reservoirs in this region
Source: PLoS Negl Trop Dis. 2026 Jan 27;20(1):e0013946. doi: 10.1371/journal.pntd.0013946 (PMC12863672; doi:10.1371/journal.pntd.0013946)
Supplement: S1 Table — Estimates from a mixed effects model with DENV PRNT80 seropositivity as the outcome and monkey age, species, and year of collection as mixed effects with troop (same collection date and site) as the random effect. (DOCX) [file pntd.0013946.s003.docx]

Table S1 Mixed effects logistic regression for DENV seropositivity. Estimates from a mixed effects model with DENV PRNT_80_ seropositivity as the outcome and monkey age, species, and year of collection as mixed effects with troop (same collection date and site) as the random effect.

| Covariate | Odds Ratio (95% CI) |
| --- | --- |
| Age | 1.46 (1.32,1.60) |
| Species (vs. *Chlorocebus sabaeus*) |  |
| *Papio* *papio* | 0.17 (0.07,0.39) |
| *Erythrocebus* *patas* | 0.21 (0.07,0.64) |
| Collection Year (vs. 2010) |  |
| 2011 | 1.14 (0.42,3.07) |
| 2012 | 0.89 (0.33,2.41) |
| Month captured (vs. January) |  |
| February | 0.49 (0.08,2.99) |
| March | 0.89 (0.24,3.34) |
| April | 1.46 (0.34,6.18) |
| May | 1.52 (0.31,7.56) |
| December | 0.81 (0.10,6.33) |
| Random Effect | Estimate (95% CI) |
| Random Effect Standard Deviation - Troop | 0.51 (0.00,0.65) |
| Intraclass Correlation Coefficient | 0.074 |
