# Supplementary material for: Seroprevalence of dengue virus antibodies among multiple species of non-human primates in Senegal suggests that sylvatic dengue virus is maintained in non-primate reservoirs in this region
Source: PLoS Negl Trop Dis. 2026 Jan 27;20(1):e0013946. doi: 10.1371/journal.pntd.0013946 (PMC12863672; doi:10.1371/journal.pntd.0013946)
Supplement: S2 Table — The age distribution is assumed to be exponentially structured with rates equal to the mean lifespans reported in this study. (DOCX) [file pntd.0013946.s004.docx]

Table S2. Estimates of *R_0_* for each NHP species by year using varying age structure assumptions, and using PRNT_50_ seropositivity to define prior infection. The age distribution is assumed to be exponentially structured with rates equal to the mean lifespans reported in this study.

| Year | *C. sabaeus* | *P. papio* | *E. patas* |
| --- | --- | --- | --- |
|  | *R_0_* (95% CI) | *R_0_* (95% CI) | *R_0_* (95% CI) |
| 2010 | 4.01 (2.78,7.59) | 3.90 (2.89,5.76) | 2.31 (1.45,9.15) |
| 2011 | 8.49 (5.34,164.29) | 3.04 (2.60,3.62) | NA |
| 2012 | 20.48 (8.28,280.14) | 2.39 (2.06,2.83) | 3.45 (2.50,5.57) |
