# Supplementary material for: Seroprevalence of dengue virus antibodies among multiple species of non-human primates in Senegal suggests that sylvatic dengue virus is maintained in non-primate reservoirs in this region
Source: PLoS Negl Trop Dis. 2026 Jan 27;20(1):e0013946. doi: 10.1371/journal.pntd.0013946 (PMC12863672; doi:10.1371/journal.pntd.0013946)
Supplement: Attachment — Submitted filename: Dengue NHP serosurvey - Response to Reviewers.docx [file pntd.0013946.s006.docx]

We very much would like to thank the reviewers for their fair and thorough reviews of out work. The comments have been extremely helpful in revising the paper.

In overview, the main changes made are:

1. Clarified throughout the manuscript that our serologic assays and force-of-infection models reflect exposure to sylvatic DENV-2 only, and expanded the Serology Methods to specify the DENV-2 strain used, the full PRNT protocol, and the PRNT50/PRNT80 thresholds that define seropositivity.
2. Added YFV PRNT results and an explicit discussion of flavivirus cross-reactivity, the potential for spillback of human-endemic DENV-2 into non-human primates, and the absence of ZIKV PRNTs as key limitations.
3. Incorporated empirical troop size summaries and behavioral/ecological differences among the three primate species to interpret species-specific DENV-2 forces of infection.
4. Streamlined and refocused the Discussion and terminology to avoid over-generalization, emphasizing that our inferences pertain to sylvatic DENV-2 in three non-human primate species in southeastern Senegal.

Please find the comments below in normal face and *the responses to each in blue italics.*

**Methods:**

Reviewer #2: Methods: I appreciate the inclusion of both PRNT50 and PRNT80.
More details are needed in the Serology section (even if in the Althouse 2018 paper). For example, which DENV serotypes were used to investigate neutralization? All? If so, is there an availability of breaking down serology by serotype?

We thank the reviewer for this helpful suggestion to clarify our serologic methods. As described in Althouse et al. 2018, PRNTs in this cohort were performed using a sylvatic DENV-2 strain that is representative of the lineage circulating in southeastern Senegal during the study period. We did not perform PRNTs against the other DENV serotypes in this cohort. In the revised Serology subsection of the Methods we now explicitly state that only DENV-2 was used in the neutralization assays and that the serologic and modeling results therefore specifically reflect sylvatic DENV-2 exposure. Because only DENV-2 was tested, we are not able to break down serologic responses by serotype.

Reviewer #3: More detail on how PRNTs were performed is required

We agree that additional detail on the PRNT protocol is important for reproducibility. In the revised manuscript we have expanded the Serology subsection of the Methods to describe the focus-reduction neutralization test protocol used: “Sera were tested for DENV-2 antibody by a plaque reduction neutralization tests (PRNT) performed in 12-well plates with confluent Vero cell cultures using 800 focus-forming units of virus. Serial dilutions of sera were mixed with an equal volume of virus and incubated for one hour at 37°C, 5% CO_2_. A 0.25 mL volume of each serum–virus mixture was then added to wells containing Vero cells and incubated for one hour at 37°C. After adsorption, 1.0 mL of 4% methylcellulose in OPTIMEM-I overlay (GIBCO BRL, Gaithersburg, MD) was added to each well and plates were incubated for four days at 37°C, 5% CO_2_. Plates were fixed with 1:1 methanol:acetone, and foci were stained with peroxidase-conjugated anti-DENV-2 antibody and counted to determine the maximum serum dilution that neutralized 50% or 80% of foci. Neutralizing antibody titers were defined as the reciprocal of the highest serum dilution resulting in a 50% or 80% reduction in foci relative to virus-only controls (PRNT_50_ and PRNT_80_, respectively), and sera with PRNT_50_ titers ≥ 1:20 or PRNT_80_ titers ≥ 1:20 were considered seropositive for prior DENV-2 infection.”

**Results**

Reviewer #2: It appears that the authors make the assumption that DENV2 is still the only DENV to circulate sylvatically in Senegal. The most recent paper cited for this is 2024, the data therein is from 2020 (with an additional non-exclusive report of a DENV2 sylvatic infection in 2021). This is a big assumption. If only DENV2 was tested, then the entire manuscript needs to be put into the context of DENV2 only.

We appreciate this important point. Our serologic assays and models are indeed specific to sylvatic DENV-2, and we have now made this explicit throughout the manuscript. In the Introduction and Methods we clarify that, to date, confirmed sylvatic dengue virus detections in southeastern Senegal have all been DENV-2, citing the most recent reports available to us (including the 2021 sylvatic DENV-2 infection noted by the reviewer). Extensive annual arbovirus surveillance by Institut Pasteur de Dakar in the Kédougou region since 1970 has not detected other DENV serotypes in the sylvatic cycle to date, and we therefore interpret our findings in the context of sylvatic DENV-2 while acknowledging that other serotypes circulate in urban human transmission cycles elsewhere in Senegal. We also now clearly state in the Methods (Serology) that only DENV-2 was used in neutralization assays in this cohort. In the Results and Discussion, we have revised the text to consistently refer to “sylvatic DENV-2” rather than “DENV” when interpreting seroprevalence and force-of-infection estimates, and we explicitly note that our inferences pertain to sylvatic DENV-2 rather than all DENV serotypes. We further note that our samples were collected between 2010-2012, before the significant surge in human-endemic DENV in Senegal.

Reviewer #3: Yes

**Conclusions**

Reviewer #2: See comments in Results.

Reviewer #3: Several sections of the discussion are tangential to the data presented and could be summarized.

***We thank the reviewer for this comment, we have addressed it by shortening the discussion.***

**Comments**

Reviewer #2: This paper provides much needed data about the distribution of dengue in West Africa. This is a straightforward yet important study. The methods are sound the results are reported appropriately. However, there is some concern with over generalization.

We thank the reviewer for the positive assessment and for highlighting the risk of over-generalization. In revising the manuscript, we carefully reviewed the Introduction, Results, and Discussion to ensure that our conclusions are framed tightly around the data we present. Specifically, we have (i) replaced several uses of “dengue” with “sylvatic DENV-2” when referring to our findings; (ii) added statements in the Discussion highlighting that our results are specific to three non-human primate species in one region of Senegal and may not generalize to other host species or geographic settings; and (iii) tempered language about broader implications for human dengue emergence, emphasizing that our data provide evidence on sylvatic DENV-2 circulation and potential, rather than definitive predictions for human outbreaks.

Reviewer #3: The authors present a neat study discussing sylvatic DENV-2 seroprevalence and transmission dynamics in three monkey species in Senegal. These data are part of a larger study that characterized the sylvatic cycle of multiple arboviruses in Senegal. Results of CHIKV serologies were presented in a prior publication. However, I do not think that it is prudent to take a similar approach for DENV-2, particularly given the high degree of cross-reactivity seen among flaviviruses seen even with neutralization tests. My main suggestion to the authors is to consider reporting the YFV and ZIKV data here, or if there was little or no cross-reactivity seen in this cohort, then to explicitly state this as a reason for not factoring this into their models. My remaining major comments are outlined below.

*We are grateful for this thoughtful summary and for the reviewer’s emphasis on flavivirus cross-reactivity. We agree that cross-reactivity between DENV, YFV, ZIKV, and other flaviviruses is a key consideration in interpreting serology results, including in some cases neutralization assays. In this cohort, YFV PRNT_90_ assays were performed as part of the larger arbovirus study; in the revised manuscript we now report the following: “*YFV PRNT_90_ assays were available for 298 NHPs in this cohort (94 *C. sabaeus*, 7 *E. patas*, and 197 *P. papio*). Among these, 176 animals had any evidence of YFV PRNT_90_ reactivity, and age-seroprevalence patterns did not demonstrate a clear increase with age (Figure S1). There was a significant association between DENV-2 and YFV seropositivity (χ^2^ test p<0.001), which may indicate common exposure or cross-reactivity or, alternatively, heterogeneity in exposure to arboviruses. A total of 67 subjects had a recorded quantitative YFV PRNT_90_ titer. However, titers among these 67 animals were generally low and did not define a clear YFV-exposed group. Given the paucity of PRNT_90_-positive animals under our prespecified criteria and the relatively small number with detectable titers, we did not attempt to jointly model YFV and DENV-2 forces of infection and instead presented the YFV results descriptively as contextual information.*”*

*In the discussion, we include cross-reactivity to ZIKV and YFV as a limitation to our interpretation: “Finally, we did not perform Zika virus (ZIKV) PRNTs in this cohort because validated ZIKV neutralization assays were not yet available at the time of sample processing and resources were prioritized toward arboviruses known to be actively circulating in the region (including DENV-2, YFV, and CHIKV). YFV PRNTs were performed only on a subset of the samples, limiting our ability to parameterize a model of cross-reactivity. As a result, we cannot directly assess potential ZIKV exposure or quantify ZIKV’s and YFV’s contribution to flavivirus cross-reactivity in our DENV-2 PRNTs. We therefore interpret our DENV-2 serologic results with caution, noting that unmeasured ZIKV or other flavivirus infections could contribute to residual cross-neutralization in a small subset of sera and overestimation of sylvatic DENV-2 FOI.”*

- I am impressed by the scale of the field collection effort. Did the authors have any strategies to ensure no repeat sampling of individuals?

We thank the reviewer for recognizing the scope of the fieldwork and for raising this important point. We have included in the description of trapping methods a discussion of methods to preclude repeat sampling, while acknowledging this possibility: “Capture sessions were organized by trapping troops at specific sites during defined time windows, and individuals were identified in the field by a combination of species, sex, estimated age class, body size, and unique physical characteristics (e.g., scars, pelage markings). Animals were released at the site of capture after sampling. While we cannot absolutely exclude the possibility of recapturing a small number of individuals across years, the geographic separation of trapping sites, the temporal spacing of campaigns, and the large populations and vast home ranges of these three species in this region together served to minimize repeat sampling. Moreover rare resampling is unlikely to materially affect our reported age-stratified seroprevalence patterns we report.”

- The parent paper (Althouse 2018) does not show which virus strains (accession number) were used for PRNT. It would be important to do so at least for DENV-2, here. Also, PRNT50 and PRNT80 thresholds for seropositivity should be clearly defined.

We appreciate this suggestion. In the revised Methods we now provide the strain designation and GenBank accession number for the DENV-2 strain (DENV-2 strain 16681, a 1964 human isolate from Thailand (GenBank Accession No. U87411.1)) used in PRNTs, and we explicitly state that this strain was isolated from the sylvatic cycle in southeastern Senegal. We also now clearly define our seropositivity thresholds: “Neutralizing antibody titers were defined as the reciprocal of the highest serum dilution resulting in a 50% or 80% reduction in foci relative to virus-only controls (PRNT_50_ and PRNT_80_, respectively), and sera with PRNT_50_ titers ≥ 1:20 or PRNT_80_ titers ≥ 1:20 were considered seropositive for prior DENV-2 infection.”

- Given the concern for cross-protection/ cross-reactivity between flaviviruses, and knowing that YFV PRNTs were done on this cohort, the authors should strongly consider reporting these data here. If there was little or no cross-reactivity, then this can be easily shown. If there was cross-reactivity, could the models be adapted to consider this? What about ZIKV? Were PRNTs performed for ZIKV and if not, why not?

We agree that presenting the YFV data and explicitly addressing flavivirus cross-reactivity strengthens the manuscript. As described above, in the revision we have added a subsection of the Results and supplementary figure describing YFV seroprevalence in this cohort, including age- and species-specific patterns. We also include a short analysis of the overlap between DENV-2 and YFV seropositivity to illustrate the extent of apparent cross-reactivity. The number of animals with discordant titers that would allow us to distinguish prior YFV versus DENV-2 infection is limited, and as a result we concluded that extending the current force-of-infection model to jointly account for cross-immunity would be underpowered and highly assumption-dependent.

We now discuss this explicitly in the Discussion as a limitation and outline how future studies with larger sample sizes and panel testing against multiple flaviviruses could enable such modeling (“Future studies using multi-flavivirus panels including ZIKV will increase our understanding not only of sylvatic DENV transmission but also of important arboviruses with spillover risk.”). As noted above, ZIKV PRNTs were not performed in this cohort due to resource constraints and prioritization of the arboviruses that were most active in the region during the sampling period; we have added a sentence explaining this and acknowledging that unmeasured ZIKV exposure could contribute to residual serologic cross-reactivity.

- In the discussion the authors note significant variability in trends among species, attributing this to troop size. Could they comment on usual troop sizes and behaviors of these different species?

We thank the reviewer for this suggestion. In the Discussion we now provide typical troop size ranges and salient behavioral/ecological characteristics for the three species studied, drawing on published primatology sources. We discuss how differences in group size, ranging behavior, and arboreality with vector exposure could plausibly contribute to the species-specific seroprevalence and force-of-infection patterns we observed.

- Is there any concern for spillback in these NHPs and could the DENV-2 seropositivity reflect this rather than true sylvatic DENV-2 infection? While I am not suggesting the authors perform PRNTs against different DENV-2 strains here, I think a brief mention of this potential limitation is warranted.

We agree that potential spillback from human transmission cycles into non-human primates is an important consideration. We have added a paragraph to the Discussion noting that we cannot definitively distinguish antibodies resulting from strictly sylvatic DENV-2 transmission versus occasional spillback of human-endemic DENV-2 into NHPs. However, we note that (i) the sampling locations are in forested areas distant from locations where endemic DENV has been detected and where the known sylvatic DENV-2 cycle is active; (ii) the temporal patterns in seroprevalence and force-of-infection estimates align with periods of documented sylvatic amplification; and (iii) there is limited evidence of sustained human dengue transmission in the immediate vicinity of our trapping sites during the study period. We now explicitly acknowledge that some fraction of seropositivity could reflect spillback and that this would not change the overall conclusion that NHPs in these areas are frequently exposed to DENV-2 but could influence the relative contribution of sylvatic versus human-derived virus.

- The discussion is too long – for instance the paragraphs on amplification hosts (Lines 288-303), Aedes vectors (317-332), and changing land-use (Lines 359-368) present more like a review than a discussion of this study’s findings, and this ends up diluting the helpful discussion of other seroprevalence studies and the CHIKV findings in this cohort. Can the authors condense these paragraphs into a few salient points?

We appreciate this guidance to streamline the Discussion. In response, we have substantially condensed the paragraphs on amplification hosts, Aedes vectors, and land-use change. Rather than providing extended background, we now: (i) briefly summarize the key points from the broader literature that are directly relevant to interpreting our data; (ii) focus on how our findings on sylvatic DENV-2 seroprevalence and FOI fit with prior seroprevalence studies and with CHIKV findings in this same cohort; and (iii) remove tangential material that read more like a general review. The revised Discussion is shorter and more tightly focused on the implications and limitations of our study.

- Line 370-371: “By… highlighting how easily the virus can cross species and ecological boundaries,” I don’t think this is truly shown in this study?

We thank the reviewer for pointing out this overstatement. We have removed the phrase suggesting that our data directly demonstrate how “easily” DENV-2 crosses species and ecological boundaries. The revised text now more cautiously states that our findings illustrate that multiple non-human primate species in this region are exposed to sylvatic DENV-2 and that such exposure, together with known vector distributions, underscores the potential for viral movement among species, without implying that our data quantify the ease of such transmission.

- I cannot find the dataset using the provided doi, could the authors check that this is correct?

We appreciate the reviewer bringing this to our attention. We identified an error in the dataset DOI cited in the Data Availability statement. The DOI has now been corrected to [https://doi.org/10.5281/zenodo.15786080], which links to the full serologic and modeling dataset used in this analysis. We have updated the Data Availability section accordingly and verified that the DOI resolves properly.

Minor comments:
Typo in Line 181: “estimated using by maximizing the likelihood” - remove “using”

We have corrected this typographical error in the Methods.

Correct “DENV” with “DENV2” throughout as relevant (e.g., Lines 218, 219, 223, etc)

We have carefully reviewed the manuscript and replaced “DENV” with “DENV-2” where the text refers specifically to the sylvatic DENV-2 virus studied here, while retaining the more general term “dengue” or “DENV” when referring to the broader literature or to dengue viruses as a group.
